# Robotic-assisted proximal gastrectomy with right-sided overlap and single-flap valvuloplasty

**DOI:** 10.3389/fsurg.2026.1794746

**Published:** 2026-05-20

**Authors:** Wei Liu, Shuawei Chen, Kai Wang, Haixiao Fu, Tengteng Li, Hao Liu, Wei Fu, Xuan Zhang

**Affiliations:** Department of General Surgery, The Affiliated Hospital of Xuzhou Medical University, Xuzhou, China

**Keywords:** anastomotic complications, gastroesophageal reflux disease, proximal gastrectomy, right-sided overlap single-flap valvuloplasty, robotic-assisted surgery

## Abstract

**Background:**

The incidence of proximal gastric cancer is increasing. Traditional total gastrectomy compromises nutritional status, while proximal gastrectomy increases the risk of gastroesophageal reflux disease (GERD). To address the reflux issues associated with proximal gastrectomy, robotic-assisted right-sided overlap single-flap valvuloplasty (ROSF) has been developed to overcome the technical challenges of anti-reflux anastomosis. This study evaluates the safety, feasibility, and anti-reflux efficacy of ROSF.

**Methods:**

A descriptive case series included 20 patients undergoing robotic-assisted proximal gastrectomy with ROSF for Siewert II/III tumors between April 2023 and April 2024. Outcomes included operative metrics, complications (Clavien-Dindo classification), reflux severity assessed by the GERD scale and Los Angeles classification, and follow-up data.

**Results:**

All procedures were completed robotically without conversion. One anastomotic stricture (5.0%) occurred and was resolved with endoscopic dilation. No reflux esophagitis, anastomotic leaks, or postoperative mortality occurred. The mean lymph node yield was 31 ± 6.8, with 14.7 ± 4.3 nodes harvested from the superior margin of the pancreas. GERD scores comparing preoperative (2.4 ± 1.0) and 6-month postoperative (2.7 ± 0.6; *P* = 0.148) values showed no significant worsening. Endoscopy confirmed intact anastomoses and normal mucosa at the anastomotic site.

**Conclusion:**

Robotic-assisted ROSF is a safe and effective technique for proximal gastrectomy, minimizing reflux and anastomotic complications. Robotic-assisted ROSF is a safe and effective technique for proximal gastrectomy, with favorable short-term outcomes in terms of technical feasibility, anastomotic safety, reflux control, and short-term nutritional stability. Its technical precision in anti-reflux reconstruction and simplified workflow support broader adoption.

## 1 Introduction

In recent years, while the incidence and mortality rates of gastric cancer have decreased, cancers of the upper stomach and gastroesophageal junction have increased. Specifically, adenocarcinomas at the gastroesophageal junction now represent 35.7% of all gastric tumors, an increase from 22.3% in prior years (1). The Surveillance, Epidemiology, and End Results (SEER) program indicates a 2.5-fold increase in proximal gastric cancer incidence over the past 35 years (2), corroborated by a 7.3% rise reported by the National Cancer Center Hospital in Japan from the 1960s to the early 21st century. Surgical management for Siewert type II and III adenocarcinomas typically involves total gastrectomy (TG) with D_2_ lymphadenectomy, which effectively removes potentially metastatic lymph nodes but carries risks of nutrient malabsorption and a reduced capacity for food intake (3–6). Conversely, proximal gastrectomy offers lower postoperative complication rates and better nutritional outcomes but compromises the gastroesophageal junction's physiological anti-reflux function, potentially worsening gastroesophageal reflux disease (GERD) due to injury to the vagus nerve (7). Studies show that the incidence of reflux esophagitis after proximal gastrectomy ranges between 21.8% and 71.6% (8, 9). Thus, techniques that reconstruct the gastroesophageal valve mechanism have gained attention. Kamikawa et al. introduced double flap plasty in 2001 (10), which reduced severe reflux esophagitis despite being technically challenging and associated with a high incidence of anastomotic stricture (up to 30%). The modified side overlap with fundoplication (mSOFY) simplifies this technique but shows variable effectiveness among patients and carries risks of leaks. In 2021, Wu proposed the right-sided overlap with single flap valvuloplasty (ROSF), combining the advantages of dual flap anastomosis and mSOFY (11). Although ROSF has gained popularity, it faces technical challenges, especially in patients with specific anatomical conditions. Robotic-assisted surgery enhances this approach with advanced features such as high-definition visualization and tremor filtering, facilitating smaller incisions and improved suturing (12). Our center has begun robotic-assisted ROSF after completing 10 prior laparoscopic cases, to streamline surgical suturing and reduce complications. This study evaluates the safety and postoperative outcomes of robotic ROSF, specifically focusing on the incidence of reflux esophagitis.

## 2 Materials and methods

### 2.1 Study subjects

This study employed a descriptive case series methodology. The inclusion criteria for patients were as follows: (1) Preoperative endoscopic biopsy and imaging studies confirming the diagnosis of esophagogastric junction cancer or upper gastric cancer; (2) Tumor diameter less than 4 cm, with no evidence of metastasis; (3) Preoperative clinical staging classified as cT_1−3_N_1_M_0_; (4) Preservation of more than 50% of gastric volume while ensuring radical resection of the proximal stomach; (5) Patients who underwent robotic-assisted proximal gastrectomy with right-sided single flap anastomosis.

Exclusion criteria included: (1) Patients who had received neoadjuvant chemotherapy prior to surgery; (2) Patients with severe cardiopulmonary conditions or other surgical contraindications; (3) Patients with poor nutritional status who could not tolerate surgery; (4) Patients with incomplete or insufficient clinical data.

The study cohort includes 20 patients who consecutively underwent robotic-assisted ROSF surgery at Xuzhou Medical University Affiliated Hospital from April 2023 to April 2024. Basic demographic information is summarized in Table 1. This study received approval from the Ethics Committee of Xuzhou Medical University Affiliated Hospital (Approval No. XYFY 2022-JS008-08). Prior to the procedure, we obtained informed consent from all patients and their families after thoroughly explaining this technique.

**Table 1:** The baseline demographic and clinical characteristics of the patients.

Patient characteristics	(*n* = 20)
Gender, No. (%)	
Male	15 (75%)
Female	5 (25%)
Age	62 (35 ∼ 75)
BMI (kg/m^2^)	25.3 (23.3 ∼ 28.6)
NRS2002 (score)	2.05 ± 1.31
Prior abdominal surgery [*n* (%)]	0 (0%)
ASA score, No. (%)	
Ⅰ	16 (80%)
Ⅱ	3 (15%)
Ⅲ	1 (5%)
ECOG (score)	
0	15 (75%)
1	4 (20%)
2	1 (5%)
Histological type of carcinoma [*n* (%)]	
Adenocarcinoma	17 (85%)
Mucinous adenocarcinoma	2 (10%)
Signet ring cell carcinoma	1 (5%)
Location of the tumor [*n* (%)]	
Gastroesophageal junction	11 (55%)
Proximal stomach	9 (45%)
cTNM stage[*n* (%)]	
Ⅰ	13 (65%)
Ⅱ	7 (35%)
Ⅲ	0 (0%)
Preoperative CEA [ng/mL, M (Range)]	4.12 (0.8 ∼ 14.1)
Preoperative CA72-4 [U/mL, M (Range)]	9.27 (0.6 ∼ 16.8)
Neoadjuvant chemotherapy, No. (%)	0 (0%)

### 2.2 Surgical methodology

All surgical procedures in this study were carried out by the same surgical team. Patients were positioned supine with their legs separated and inclined, with the head elevated and feet lowered. A 2 cm incision was made 2 cm above the umbilicus to establish pneumoperitoneum, maintaining an intra-abdominal pressure of 10 ∼ 12 mmHg. Following successful establishment of pneumoperitoneum, an 8 mm trocar was inserted as the observation port, allowing exploration of the abdominal cavity.

**Figure 1:**
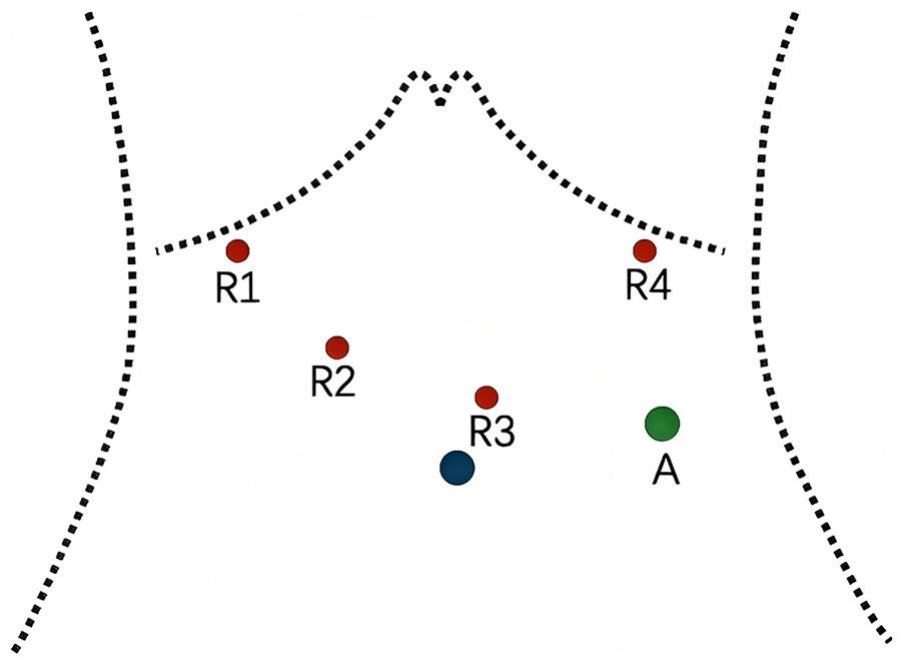
Schematic representation of trocar placement configuration.

After confirming suitability for robotic-assisted radical gastric cancer surgery, a “5-port” technique was utilized for trocar placement (Figure 1). The placements were as follows: an 8 mm trocar was inserted at the umbilical incision for observation; an 8 mm trocar was positioned under the left anterior axillary line at the costal margin as the main operating port for the fourth robotic arm; a 12 mm trocar was inserted 2 cm below the left mid-clavicular line as an assistant operating port; an 8 mm trocar was placed 2 cm above the right mid-clavicular line as the operating port for the second robotic arm; and an 8 mm trocar was inserted under the right anterior axillary line at the costal margin as the operating port for the first robotic arm. The distance between adjacent trocars was maintained at greater than 8 cm apart.

The da Vinci Xi robotic system was then installed and secured, with the robotic arms positioned at the patient's head side, aligned with the midline of the patient's body. The observation port was connected to the third robotic arm, and the camera was inserted. Once the surgical field was established, the arms adopted a “hugging” configuration: the camera arm was centered, while the instrument arms were appropriately abducted to prevent collision, ensuring that the numerical displays on the arms faced forward. The first robotic arm was connected to a curved grasper, the second arm to a fenestrated bipolar forceps, and the fourth arm to a Maryland bipolar forceps.

### 2.3 Surgical procedures

Routine exploration of the abdominal cavity was conducted to assess for any metastatic lesions on the peritoneum, omentum, and organ surfaces. The greater omentum was lifted, and an incision was made along the avascular area of the gastrocolic ligament on the left side of the midpoint of the greater curvature of the stomach, allowing entry into the omental bursa. The left gastroepiploic vessels were clamped and divided, followed by the division of several short gastric vessels and the gastro-splenic ligament, thereby exposing the left side of the cardia. A dissection of the lesser omentum was performed from right to left along the inferior margin of the liver, extending to the right side of the cardia. Next, a longitudinal incision was made on the right side of the cardia along the lesser curvature of the stomach down to the angular incisure, allowing for the mobilization of the lower esophagus. The esophageal transection line and the gastric transection line were marked based on intraoperative endoscopy. The specimen was subsequently excised, and the margins were sent for rapid frozen pathological examination. The procedure involved a complete robotic-assisted reconstruction of side-overlap fundoplication (ROSF), outlined as follows.

**Figure 2:**
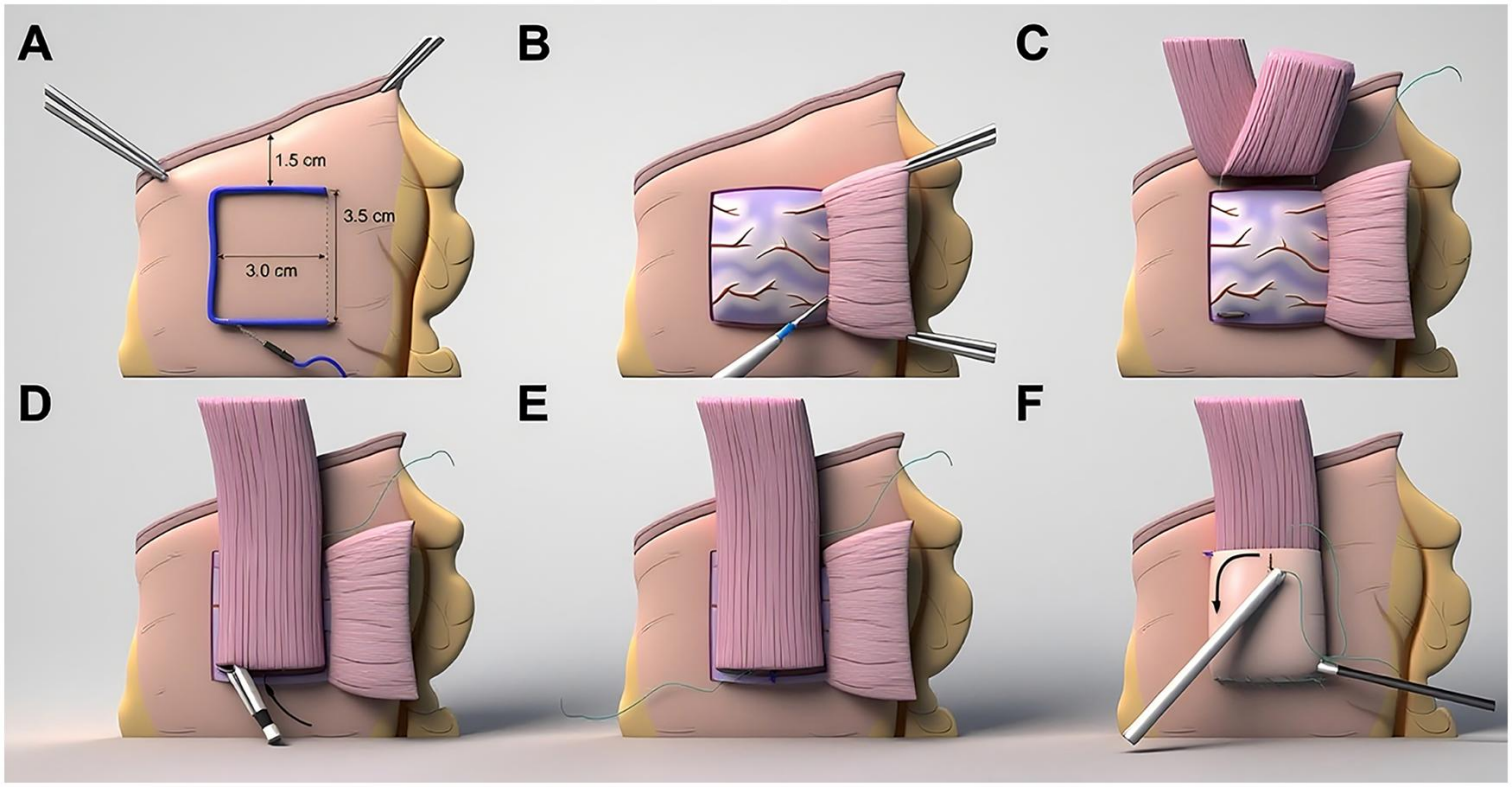
Schematic of right-sided overlap single-flap valvuloplasty (ROSF) procedure.

**Figure 3:**
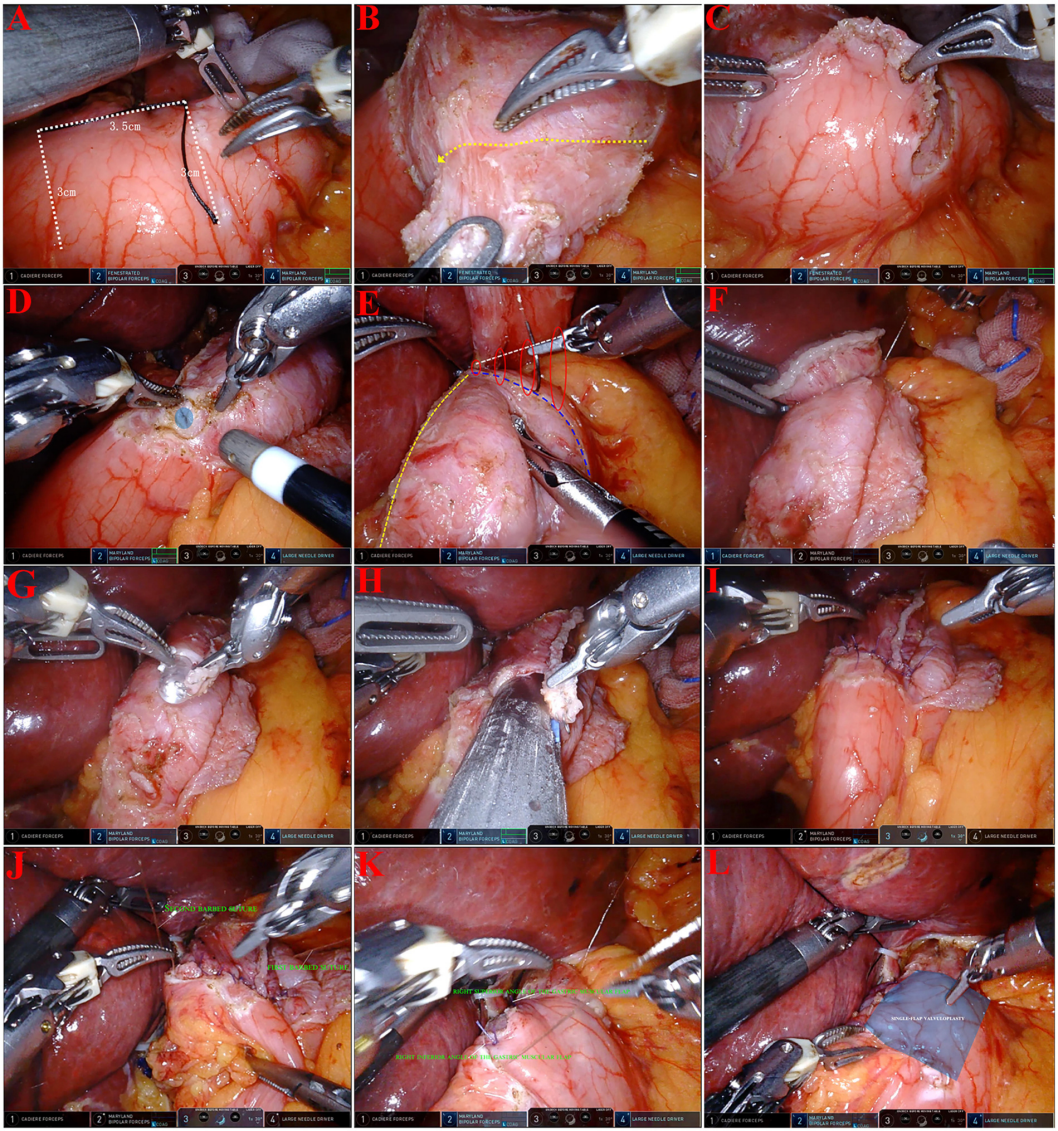
Surgical procedures for ROSF. **(A)** Marking the boundaries of the single-flap valvuloplasty (The white dotted line delineates the boundary for the construction of the single-layer). **(B)** Freeing the right flap single-flap valvuloplasty (The yellow-dashed line indicates the interlayer space developed during seromuscular-submucosal dissection). **(C)** Completing the freeing of the ROSF. **(D)** Opening gastric mucosa at the midpoint of the bottom edge of the mucosal area of the gastric muscle flap, 0.5 cm from the bottom edge, 0.5 cm × 0.5 cm in size. **(E)** The first barbed line starts from the right to the left and the anastomotic posterior wall is reinforced with a suture; The yellow dashed line delineates the right mucosal margin; the blue dashed line demarcates the cephalic mucosal boundary of the gastric muscle flap; the white dashed line indicates the dorsal esophageal contour, positioned 3 cm distal to the esophageal stump. **(F)** Completing the Reinforcement suture of the posterior wall of the anastomosis. **(G)** Open the right corner of the oesophageal stump and insert the gastric tube out as a guide. **(H)** Straight line cutting closure to anastomose the right wall of the oesophagus to the longitudinal midline of the gastric muscle flap mucosa. **(I)** Intermittent suture of the common opening with 3 ∼ 5 stitches. **(J)** The second barbed line to close the common opening. **(K)** Suture of the right upper corner of the gastric muscle flap and the right lower corner in the gastric plasma myotome on the right boundary of the gastric muscle flap mucosal area. **(L)** Completion of ROSF.

A rectangular area measuring 3.0 cm in width and 3.5 cm in height was marked 1.5 cm below the closure line of the remaining stomach (Figures 2, 3). Using Maryland bipolar forceps, dissection was performed between the submucosal and muscular layers to create a right-sided single seromuscular flap, taking care to avoid damaging the submucosal blood vessels. Five points were marked with methylene blue along the serosa at the upper margin of the mucosal window. The blue-marked points were then sutured to the posterior wall of the esophagus 5 cm above the esophageal transection line using five sutures. After pushing the remaining stomach posteriorly into the mediastinum, the sutures were tightened to secure the esophageal posterior wall to the anterior wall of the stomach.

An incision was made at the lower right corner of the mucosal window to access the gastric mucosa, and a gastric tube was inserted to confirm mucosal integrity. A linear cutting stapler was then used to anastomose the posterior wall of the esophagus and the anterior wall of the gastric mucosal window on the right side, inserting the stapler to a depth of 3 cm. Initially, a 4–0 suture was used for intermittent closure of the common opening. A continuous suture with a 3–0 barbed suture (the second barbed suture) was performed from left to right to secure the esophagogastric mucosal side-overlap anastomosis, ensuring reliable suturing of the esophageal mucosa. Following this, the lower edge of the barbed suture used to close the common opening was employed to suture the edge of the seromuscular flap to the gastric wall with 3 to 4 stitches.

An assistant applied downward pressure to the stomach, exposing the site for securing the esophagogastric junction. The upper right corner of the seromuscular flap was sutured and tied at this point. The first barbed suture, which fixed the gastric wall and the posterior wall of the esophagus, was utilized to suture the upper edge and right edge of the seromuscular flap. Starting from the left side of the upper edge of the flap, four sutures were placed to secure the flap to the esophagus, ensuring that the sutures were not overly tight. At this stage, the upper left corner of the mucosal window was at risk of bulging; thus, the seromuscular flap was adequately used to cover this area to prevent protrusion. At the upper right corner of the seromuscular flap, the barbed suture was directed to the right edge of the flap, enabling continuous suturing to the seromuscular layer of the gastric wall on the right side of the mucosal window. Given that the common opening was located at the lower right corner of the mucosal window, precise suturing of this area of the flap was crucial to avoid leakage at the anastomosis site. Upon completion of the anastomosis, an endoscopic examination was conducted to ensure the integrity of the anastomosis, checking for active bleeding, stenosis, and gas leakage.

### 2.4 Efficacy evaluation criteria and follow-up

#### 2.4.1 Observational indicators

1. Surgical Duration and Digestive Reconstruction Time: The total surgical duration for each patient was recorded in chronological order.
2. Intraoperative Blood Loss and Number of Lymph Nodes Cleared: The volume of blood loss during surgery and the number of lymph nodes excised were documented.
3. Postoperative Complications: The incidence of postoperative complications within 30 days was assessed, including incision infections, pulmonary infections such as pneumonia, pleural effusion, intra-abdominal hemorrhage, lymphatic leakage, anastomotic leakage, anastomotic stricture, and postoperative gastroparesis.
4. Postoperative Recovery: Recovery metrics were evaluated, including length of hospital stay, time to first flatus, symptoms of gastroesophageal reflux, and diagnosis of esophagitis, with upper gastrointestinal imaging conducted before and shortly after discharge.
5. Postoperative nutritional outcomes were also evaluated using body weight, BMI, hemoglobin, albumin, and prealbumin at 3 and 6 months after surgery.

#### 2.4.2 Evaluation criteria

Postoperative complications were assessed using the Clavien-Dindo classification system. Esophagitis was diagnosed through upper gastrointestinal imaging, and the extent of lesions was evaluated using the Los Angeles classification system. The severity of GERD symptoms was scored using a standardized GERD scale (13). Follow-up was conducted through outpatient visits, inpatient assessments, telephone consultations, email communications, and home visits. Patients were queried regarding their postoperative dietary habits and the presence of gastroesophageal reflux symptoms; records of GERD scale scores were maintained accordingly. Follow-up assessments were scheduled monthly for the first year post-surgery.

#### 2.4.3 Statistical methods

Statistical analysis was performed using the Statistical Package for the Social Sciences (SPSS) version 22.0. Normally distributed continuous variables were expressed as mean ± standard deviation (SD), while categorical data were presented as counts and percentages. The comparison of GERD scores at three months postoperatively and preoperative values was conducted using the paired *t*-test. *P* < 0.05 was considered to indicate statistical significance.

## 3 Results

### 3.1 Perioperative conditions

All patients successfully underwent robotic surgery using the Da Vinci system, with the procedures progressing smoothly and no cases requiring intraoperative conversion to open surgery. Postoperatively, one case of anastomotic stricture occurred among 20 patients (5.0%), which was successfully treated with endoscopic dilation. Upper gastrointestinal series and endoscopy showed no signs of reflux esophagitis, and no reflux symptoms were observed. There were no instances of anastomotic leakage, anastomotic bleeding, local infection, or mortality among the patients. Additionally, there were no functional complications such as delayed gastric emptying, and no significant cases of chylous leakage were observed post-surgery. Detailed perioperative data are presented in Table 2.

**Table 2:** The perioperative clinical data of the patients.

Variable	(*n* = 20)
Type of operation, No. (%)	
Proximal gastrectomy	20 (100%)
Reconstructive surgical approaches, No. (%)	
ROSF	20 (100%)
Preoperative preparation period (min)	26.2 (20 ∼ 30)
Operative time (min)	236 ± 23.8 (210 ∼ 289)
Estimated blood loss (mL)	36 ± 19.2 (21 ∼ 89)
Blood transfusions, No. (%)	0 (0%)
Conversion to open surgery, No. (%)	0 (0%)
Days to first flatus emission after surgery (h)	2.5 ± 0.7 (1 ∼ 3)
First flow time (d)	3.5 ± 1.1 (2 ∼ 4)
Time of catheter removal (d)	1 ± 0.2 (1 ∼ 2)
Time to get out of bed (d)	1 ± 0.2 (1 ∼ 2)
Levels of amylase in peritoneal effusion (U/L)	
The first postoperative day	321 ± 121
The second postoperative day	225 ± 97
The third postoperative day	85 ± 22
Postoperative timing of abdominal drainage tube removal (d)	5.3 ± 1.5
Complications, no. (%)	
anastomotic leakage	0 (0%)
anastomotic bleeding	0 (0%)
Anastomotic stricture	1 (5%)
Gastric emptying dysfunction	0 (0%)
Wound infection	1 (5%)
Pulmonary infection	1 (5%)
Chylous leakage	0 (0%)
Clavien-Dindo grade, No. (%)	
I	3 (15%)
II	0 (0%)
III	0 (0%)
Postoperative pain grading, No. (%)	
Level 1	13 (65%)
Level 2	6 (30%)
Level 3	1 (5%)
Level 4	0 (0%)
Postoperative hospital stay (d)	7.6 (6 ∼ 9)
Hospitalization expenses (ten thousand yuan)	8.5 ± 1.3 (7.1 ∼ 9.8)
Robotics-associated costs (ten thousand yuan)	2.2 ± 0.4 (1.8 ∼ 2.6)
Mortality within 30 d of operation, no. (%)	0 (0%)

### 3.2 Postoperative pathological findings

The average distance from the tumor to the proximal margin was 1.9 ± 1.1 cm, while the average distance to the distal margin was 5.7 ± 2.1 cm. The total number of lymph nodes retrieved during the procedure was 31 ± 6.8, with an average of 3.4 ± 2.4 positive lymph nodes identified. Specifically, the average number of lymph nodes harvested from the superior pancreatic margin was 14.7 ± 4.3. Regarding postoperative pathological staging (TNM classification), the cases were classified as follows: 15 cases as Stage I, 4 cases as Stage II, and 1 case as Stage III (Table 3).

**Table 3:** The postoperative pathological characteristics.

Pathological findings	(*n* = 20)
Tumor size (cm)	2.7 ± 1.3
Distance of upper incisal margin of tumor (cm)	1.9 ± 1.1
Distal resection margin (cm)	5.7 ± 2.1
Tumour grade of differentiation	
Well differentiated	15 (75%)
Moderately differentiated	3 (15%)
Poorly differentiated	1 (5%)
Signet ring cell carcinoma	1 (5%)
No. harvested lymph nodes	31 ± 6.8 (25 ∼ 41)
Positive lymph node count	3.4 ± 2.4 (1 ∼ 6)
Dissected lymph node count at the superior pancreatic border	14.7 ± 4.3 (12 ∼ 19)
No. 5	1.64 ± 1.8
No. 7	5.80 ± 4.4
No. 8a	3.12 ± 2.1
No. 9	1.67 ± 1.1
No. 11p	1.1 ± 0.3
No. 12a	1.31 ± 0.8
pTNM stage, No. (%)	
I	15 (75%)
II	4 (20%)
III	1 (5%)
Lauren classification, No. (%)	
Intestinal type	10 (50%)
Diffuse type	2 (10%)
Mixed type	8 (40%)
Adjuvant chemo, No. (%)	5(25%)

### 3.3 Postoperative follow-up outcomes

**Figure 4:**
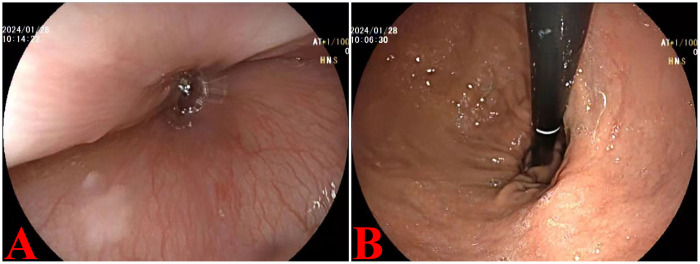
Illustrates endoscopic results one year following surgery. **(A)** An oval-shaped anastomosis was noted, with no signs of erosion present in the esophageal mucosa. **(B)** The angle of His and a pseudofornix had successfully reformed.

GERD Scale Assessment: During the postoperative follow-up period, all patients completed the GERD scale assessment monthly to evaluate their gastroesophageal reflux symptoms. The preoperative score was 2.4 ± 1.0 (mean ± standard deviation). The scores at postoperative months 1 through 6 were as follows: 3.6 ± 1.0, 3.2 ± 0.8, 3.0 ± 0.8, 2.8 ± 0.6, 2.8 ± 0.6, and 2.7 ± 0.6, respectively. There was no statistically significant difference between the GERD score at 6 months postoperatively and the preoperative score (*t* = −1.495, *P* = 0.148) (Table 4). In addition to symptom assessment, all cases underwent endoscopic re-evaluation 6 months postoperatively, which revealed normal mucosal findings (Figure 4). During postoperative follow-up, one patient received proton pump inhibitor therapy. However, this patient showed no endoscopic evidence of reflux esophagitis and no significant worsening in GERD-related evaluation.

**Table 4:** The GERD questionnaire score.

GerdQ score	*n* = 20
Preoperative Scoring	2.4 ± 1.0
1 Month Postoperative	3.6 ± 1.0
2 Month Postoperative	3.2 ± 0.8
3 Month Postoperative	3.0 ± 0.8
4 Month Postoperative	2.8 ± 0.6
5 Month Postoperative	2.8 ± 0.6
6 Month Postoperative	2.7 ± 0.6

### 3.4 Nutritional outcomes

Nutritional outcomes were additionally assessed using body weight, BMI, hemoglobin, albumin, and prealbumin before surgery and at 3 and 6 months postoperatively. As shown in Table 5, no statistically significant differences were observed across the three time points in body weight (66.3 ± 6.8 vs. 65.1 ± 7.3 vs. 66.7 ± 7.2 kg, *P* = 0.73), BMI (25.3 ± 0.7 vs. 25.3 ± 0.8 vs. 25.6 ± 0.8 kg/m², *P* = 0.81), hemoglobin (124.2 ± 4.8 vs. 122.4 ± 4.6 vs. 124.0 ± 4.6 g/L, *P* = 0.44), albumin (41.5 ± 2.3 vs. 40.8 ± 2.0 vs. 42.4 ± 2.6 g/L, *P* = 0.12), or prealbumin (280.6 ± 9.2 vs. 279.1 ± 8.8 vs. 283.4 ± 8.5 mg/L, *P* = 0.31). These findings indicate that short-term postoperative nutritional status remained generally stable in this cohort.

**Table 5:** Nutritional outcomes before and after surgery.

Variable	Preop	3 mon	6 mon	*P* value	*F* value
Weight (kg)	66.3 ± 6.8	65.1 ± 7.3	66.7 ± 7.2	0.73	0.30
BMI(kg/m²)	25.3 ± 0.7	25.3 ± 0.8	25.6 ± 0.8	0.81	0.44
Hemoglobin (g/L)	124.2 ± 4.8	122.4 ± 4.6	124.0 ± 4.6	0.44	0.82
Albumin (g/L)	41.5 ± 2.3	40.8 ± 2.0	42.4 ± 2.6	0.12	2.19
Prealbumin(mg/L)	280.6 ± 9.2	279.1 ± 8.8	283.4 ± 8.5	0.31	1.18

## 4 Discussion

The incidence of gastroesophageal reflux and anastomotic leak following proximal gastrectomy ranges from 9.1% to 35.3% and 14.6% to 35.4%, respectively, significantly impacting the postoperative quality of life for patients (11, 14, 15). Currently, there are no standardized guidelines for digestive tract reconstruction and anti-reflux measures following proximal gastrectomy. In 2016, Muraoka et al. first introduced the Kamikawa anastomosis for digestive tract reconstruction after proximal gastrectomy in patients with early-stage upper gastric cancer (16). In their study, all 24 cases were completed successfully, with no postoperative occurrences of reflux esophagitis. Similarly, Kuroda et al. performed the Kamikawa anastomosis on 33 patients with early upper gastric cancer, successfully completing the procedure laparoscopically, with no reports of reflux esophagitis during one year of follow-up (17). Although the Kamikawa anastomosis has demonstrated evident anti-reflux benefits, its clinical success is accompanied by technical challenges. The primary technical difficulty in the double-layer anastomosis is the suturing process, and because the entire reconstruction procedure is performed laparoscopically, the manual suturing steps become complex and time-consuming, which demand a high level of laparoscopic suturing skill and teamwork from the surgical team. During the incision of the muscularis layer of the gastric wall, surgeons must ensure the integrity and adequate blood supply of both the seromuscular layer and the gastric mucosa. Damage to either layer may increase the risk of postoperative anastomotic leaks.

The ROSF technique employed in this study involves creating a muscle flap from the muscularis layer of the stomach wall. This flap is initiated 1.5 cm from the closure line on the anterior wall of the remaining distal stomach. Constructed from right to left, the flap has dimensions of 3 cm in length and 3.5 cm in width, with only the left edge remaining attached to the gastric wall. This flap overlay, combined with a side-overlap technique, serves an anti-reflux function, and may provide effective reflux control while maintaining an adequate blood supply to the flap and anastomosis. Furthermore, the use of a linear cutting stapler and the manual suturing of the common opening result in a more defined and circular anastomotic circumference compared to a circular stapler, thereby reducing the likelihood of anastomotic stenosis. Additionally, the well-vascularized flap reinforces the anastomosis, aided by the encapsulating effect of the muscularis layer, which may contribute to the lower incidence of anastomotic leaks observed.

The Da Vinci Surgical System provides stable retraction and high-definition 3D visualization, enhancing the precision of anatomical dissection (18). Its flexible instruments allow for exposure, dissection, and suturing at various angles, which is particularly beneficial for performing transverse and longitudinal sutures in the confined space of the esophageal hiatus, as well as for securing the mobilized distal stomach during the flap creation process (19). During the flap construction, we noted specific advantages of the Da Vinci Surgical System compared to traditional laparoscopy: First, the system allows for prolonged and stable fixation of the mobilized distal stomach while maintaining appropriate tension on the gastric wall. This stable fixation and tension are fundamental for creating high-quality flaps. Second, the Maryland bipolar forceps, with their fine tweezers-like tips, provide precise hemostasis and minimal thermal damage. They enable millimeter-level dissection between the muscularis and mucosal layers while preserving a bloodless field and minimizing thermal injury to both the mucosal layer and muscle flap. Third, the high magnification and clarity of the 3D view enable precise differentiation of the dissection plane between the muscularis and mucosal layers, facilitating the creation of quality flaps without the need for submucosal injection of methylene blue. During the anastomosis, we similarly found advantages with the Da Vinci Surgical System. When suturing the posterior wall of the esophagus to the anterior wall of the stomach, the robotic arm maintains continuous and stable tension on the esophagus, improving exposure of the posterior wall. Additionally, the rotating function of the robot's needle holder significantly reduces the difficulty of performing longitudinal sutures from the esophagus to the stomach wall. Furthermore, during the common opening suturing and flap suturing, the system allows for precision suturing from various angles.

Our short-term outcomes should be interpreted in the context of the existing literature. Conventional proximal gastrectomy without a dedicated anti-reflux reconstruction has been associated with a substantial incidence of postoperative reflux and impaired postoperative quality of life (9, 17–19). In contrast, the double-flap technique reported by Muraoka et al. and Kuroda et al. demonstrated favorable anti-reflux efficacy, although the procedure is technically demanding and may be associated with anastomotic stricture in some patients (16, 17). Peng et al. also reported encouraging early outcomes for laparoscopic ROSF, supporting the concept that flap-based overlap reconstruction may reduce reflux while maintaining an acceptable safety profile (13). More recently, Yamashita et al. described modified side-overlap esophagogastrostomy (mSOFY) as another antireflux reconstruction with favorable short-term outcomes (20), while Kuroda et al., in the prospective multicenter lD-FLAP Study, further confirmed the anti-reflux efficacy of laparoscopic double-flap reconstruction with acceptable rates of leakage and stricture (21). Against this background, the absence of reflux esophagitis and anastomotic leakage in our series, together with only one anastomotic stricture (5.0%), appears favorable and generally consistent with the outcomes reported for other dedicated anti-reflux reconstructions after proximal gastrectomy. In addition, the GerdQ score did not significantly worsen at 6 months compared with the preoperative baseline, supporting the preliminary anti-reflux efficacy of robotic-assisted ROSF in this cohort.

At the same time, our findings should not be overinterpreted. The present study was a single-center, single-arm descriptive case series with a relatively small sample size. Most importantly, no control group was included, such as patients undergoing total gastrectomy, double-tract reconstruction, double-flap reconstruction, or mSOFY. Therefore, although the short-term results of robotic-assisted ROSF appear encouraging, this study does not permit definitive comparative conclusions regarding the superiority of ROSF over other established reconstruction methods. Any comparison with the published literature remains indirect and should be interpreted cautiously because of differences in patient selection, tumor stage, surgical indication, follow-up duration, and assessment tools across studies.

Several additional limitations should also be acknowledged. First, the follow-up period in the present study was relatively short, and the long-term durability of the anti-reflux effect remains uncertain. Second, although endoscopic and symptom-based assessments suggested satisfactory reflux control, broader postoperative functional outcomes and quality-of-life measures were not comprehensively evaluated. Third, although short-term nutritional parameters, including body weight, BMI, hemoglobin, albumin, and prealbumin, remained stable during follow-up, the limited sample size and relatively short follow-up period restrict conclusions regarding the long-term functional and nutritional advantages of this procedure beyond reflux prevention. Future studies should therefore include longer follow-up, standardized quality-of-life instruments, detailed nutritional endpoints, and ideally prospective comparative cohorts or randomized comparisons against other reconstruction techniques.

In summary, robotic-assisted ROSF achieved encouraging short-term outcomes in this case series, with excellent technical feasibility, a low rate of anastomotic complications, and favorable preliminary anti-reflux results. Nevertheless, given the absence of a control group, the present findings should be regarded as preliminary. Larger multicenter prospective studies with appropriate comparator arms are required to clarify whether robotic-assisted ROSF offers meaningful advantages over other established reconstruction strategies after proximal gastrectomy.

## Data Availability

The raw data supporting the conclusions of this article will be made available by the authors, without undue reservation.
